# Human γδ T Cell Receptor Repertoires in Peripheral Blood Remain Stable Despite Clearance of Persistent Hepatitis C Virus Infection by Direct-Acting Antiviral Drug Therapy

**DOI:** 10.3389/fimmu.2018.00510

**Published:** 2018-03-16

**Authors:** Sarina Ravens, Julia Hengst, Verena Schlapphoff, Katja Deterding, Akshay Dhingra, Christian Schultze-Florey, Christian Koenecke, Markus Cornberg, Heiner Wedemeyer, Immo Prinz

**Affiliations:** ^1^Institute of Immunology, Hannover Medical School, Hannover, Germany; ^2^Department of Gastroenterology, Hepatology and Endocrinology, Hannover Medical School, Hannover, Germany; ^3^Institute of Virology, Hannover Medical School, Hannover, Germany; ^4^Department of Hematology, Hemostasis, Oncology and Stem Cell Transplantation, Hannover Medical School, Hannover, Germany; ^5^Department of Gastroenterology and Hepatology, Essen University Hospital, Essen, Germany

**Keywords:** γδ T cells, chronic hepatitis C virus, TRG, TRD, next-generation sequencing, direct-acting antivirals

## Abstract

Human γδ T cells can contribute to clearance of hepatitis C virus (HCV) infection but also mediate liver inflammation. This study aimed to understand the clonal distribution of γδ T cells in peripheral blood of chronic HCV patients and following HCV clearance by interferon-free direct-acting antiviral drug therapies. To this end, γδ T cell receptor (TCR) repertoires were monitored by mRNA-based next-generation sequencing. While the percentage of Vγ9^+^ T cells was higher in patients with elevated liver enzymes and a few expanded Vδ3 clones could be identified in peripheral blood of 23 HCV-infected non-cirrhotic patients, overall clonality and complexity of γδ TCR repertoires were largely comparable to those of matched healthy donors. Monitoring eight chronic HCV patients before, during and up to 1 year after therapy revealed that direct-acting antiviral (DAA) drug therapies induced only minor alterations of TRG and TRD repertoires of Vγ9^+^ and Vγ9^−^ cells. Together, we show that peripheral γδ TCR repertoires display a high stability (1) by chronic HCV infection in the absence of liver cirrhosis and (2) by HCV clearance in the course of DAA drug therapy.

## Introduction

The majority of hepatitis C virus (HCV) infection results in chronicity and only 10–50% of cases are cleared in the acute phase (1, 2). Failing cytotoxic T cell activity due to exhaustion of expanded T cells causes chronic viral persistence and continuous activation of liver-infiltrating lymphocytes progressively leading to liver cirrhosis and development of hepatocellular carcinoma (3–5). However, the contribution of γδ T cells to HCV control is largely unknown.

γδ T cells are innate immune cells expressing a T cell receptor (TCR) consisting of a γ- and a δ-chain, each composed of a variable (V), diversity (D), and joining (J) gene segment generated by “VDJ recombination.” The random rearrangement of different gene segments creates a high clonal diversity, which is particularly reflected in the junctional regions (CDR3 sequence) of TCR chains. γδ T cells can be classified based on their expressed Vγ or Vδ chains functionality and distribution within the body. The Vγ9JP^+^Vδ2^+^ subset is the main population of γδ T cells within the peripheral blood of most adult healthy individuals (6). Vγ9JP^+^Vδ2^+^ cells are activated through small phosphoantigens, like microbial-derived HMB-PP or host-derived isopentyl pyrophosphate (IPP) (7, 8) and are involved in anti-cancer surveillance, pathogen clearance, or inflammatory diseases (9). By contrast, the identity of molecules activating non-Vγ9JP^+^Vδ2^+^ cells is largely unknown. Nevertheless, a few studies revealed that these could be stress molecules exposed by virus-infected or tumor cells (10–14). Overall, non-Vγ9JP^+^Vδ2^+^ γδ T cells exert a high degree of antiviral and antitumor activity (15, 16), which is for instance reflected in the expansion of Vδ1^+^ γδ T cells in response to viral infection in immunocompromised patients, stem cell transplant recipients, and during pregnancy (17–21).

The functional role of different γδ T cell populations in HCV persistence and associated liver malignancies remains to be understood. *Per se*, γδ T cells are enriched not only in liver tissues of healthy persons but also in patients with hepatitis infections (22). Hepatic γδ T cell populations express the NK-cell marker CD56, the liver-homing marker CD161, produce INF-γ, and demonstrate an effector/memory phenotype (23–25). Especially, chronic HCV patients with liver cirrhosis display elevated γδ T cell numbers and their cytokine production and cytotoxicity was suggested to play a role in inflammatory necrotic processes (26–29). Studies analyzing matched blood and liver specimens from patients with chronic liver diseases indicated that Vδ2^+^ and/or Vδ1^+^ can infiltrate the liver (25, 28). Increased Vδ1^+^ cell frequencies in liver transplant recipient were associated with high viral loads (HCV, CMV) (30). Likewise, patients infected with only HCV, or co-infected with HIV undergoing active antiretroviral therapy (HAART), had elevated Vδ1^+^ γδ T cells in the blood and liver, which was linked to liver inflammation (28, 31). Of note, HAART therapy did not restore intrahepatic Vδ1^+^ T cells to normal levels within HCV/HIV co-infected patients (31). Especially, Vγ9JP^+^Vδ2^+^ cells have been revealed to inhibit viral replication (32). Other studies connect HCV persistence to low Vγ9JP^+^Vδ2^+^ frequencies, impaired IFN-γ production, and γδ T cell exhaustion (24, 25, 33, 34), while the cytotoxicity and continuous activation of Vγ9JP^+^Vδ2^+^ during chronic HCV infection contributes to liver inflammation and cirrhosis (24). The therapeutic application of zoledronate to activate Vγ9JP^+^Vδ2^+^ γδ T cells through cellular accumulation of IPP was suggested as a strategy to apply γδ T cells to inhibit viral replication during interferon-based therapies (32, 34, 35).

Over the past few years, conventional HCV therapy based on PEG-INFα/ribavirin has been replaced by direct-acting antiviral (DAA) drugs. These DAA therapies result in increased cure rates defined by virus clearance and improve liver inflammation and cirrhosis in HCV-infected patients (36–38). Effects of DAAs and HCV clearance on the restoration of different immune cell subsets including HCV-specific T cells, NK cells, and MAIT cells have been analyzed in patients (39–43). However, to the best of our knowledge, no study addressed the effect of DAA on γδ T cell composition.

Next-generation sequencing (NGS) of TCR repertoires has the advantage to monitor γδ T cell populations at the clonal level and to identify disease-related TRG (γ-chain) and TRD (δ-chain) sequences (19, 44). To understand the clonal distribution of γδ T cells in patients with chronic HCV and to investigate in the influence of DAA on γδ T cell repertoires, we used flow cytometric cell sorting and NGS to profile γδ TCRs from total as well as Vγ9^+^ and Vγ9^−^ isolated γδ T cell populations from peripheral blood.

## Results

### Healthy and Chronic HCV Patients Show Similar γδ T Cell Repertoire Complexity

**Figure 1:**
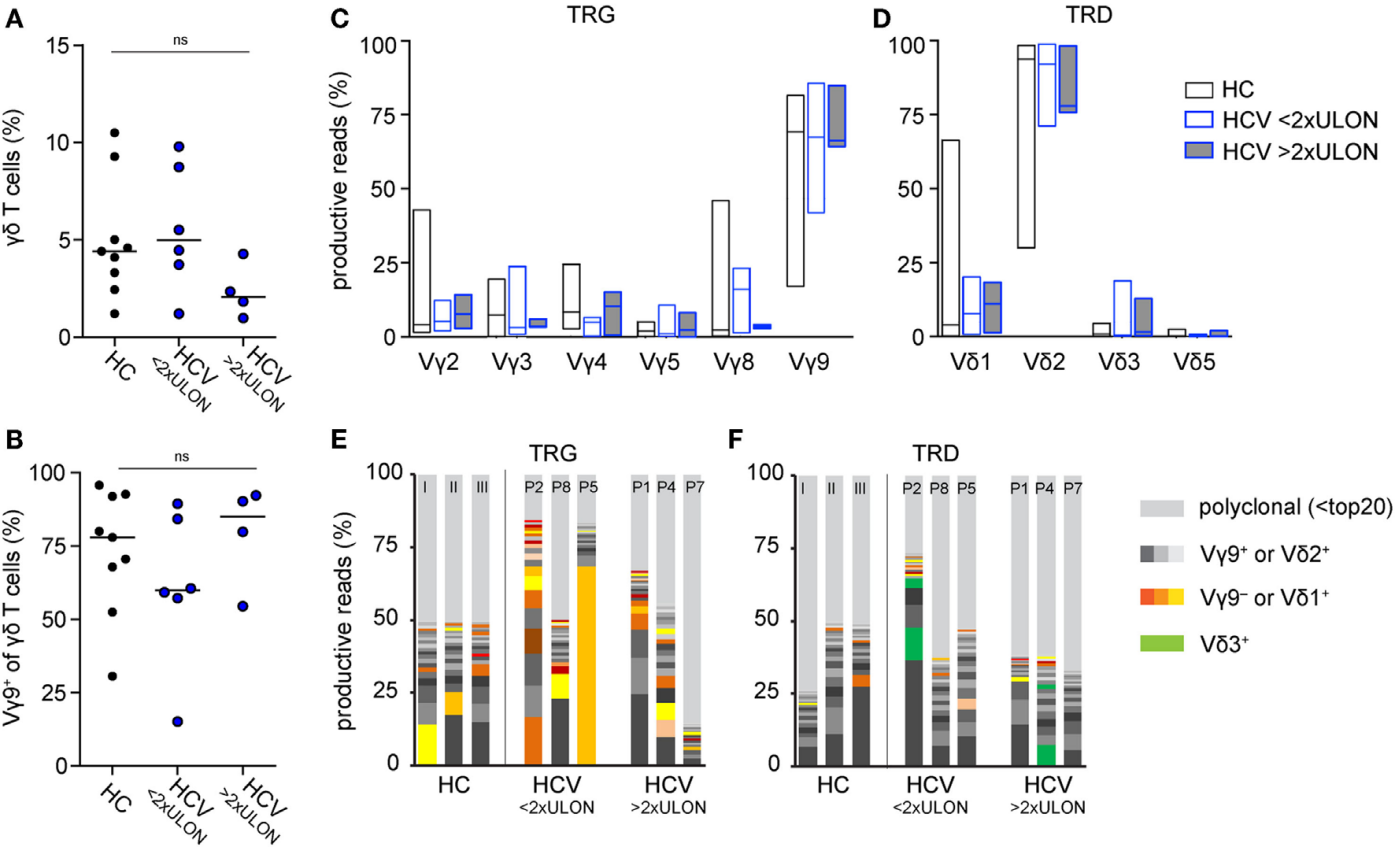
Comparison of peripheral γδ T cell repertoires in chronic hepatitis C virus (HCV) patients and healthy controls (HC). **(A,B)** Flow cytometric sorting results of 9 HC (black dots) and 10 chronic HCV patients at baseline (blue dots), which were grouped based on their alanine aminotransferase (ALT) values, are summarized in dot plots representing **(A)** total γδ T cell frequencies of CD14^−^/CD19^−^ lymphocytes and **(B)** Vγ9^+^ cell frequencies of total γδ T cells. Six patients had ALT levels two times lower than upper limit of normal (<2× ULON) and four patients had increased ALT levels (>2× ULON). Horizontal lines present median values. Data were analyzed by Mann–Whitney test. **(C,D)** Sorted γδ T cells of eight HC and eight chronic HCV patients, the latter grouped based on their ALT levels, were subjected to next-generation sequencing (NGS) analysis. Floating bars from minimum to maximum represent the distribution of functional V-genes of analyzed **(C)** TRG or **(D)** TRD repertoires. Lines represent median values. **(E,F)** The most expanded top 20 **(E)** TRG or **(F)** TRD clones are highlighted in stacked area graphs of three representative HC and six HCV patients who were stratified due to their ALT levels. Vγ9^−^ or Vδ1^+^ clones are highlighted in orange, Vγ9^+^ or Vδ2^+^ in gray, Vδ3^+^ clones in green color, and non-top 20 clones in light gray. NGS samples were normalized to the percentage of all productive sequences per sample.

We monitored peripheral γδ T cell repertoires in 10 patients with chronic HCV infections before, during, and after therapy with DAAs and in additional 13 patients at a single time point during therapeutic DAA treatments. All patients had persistent viral infection with the HCV genotype 1 (Table 1 for patients’ characteristics). Even though parameters for liver inflammation, such as alanine aminotransferase (ALT) and aspartate aminotransferase (AST) level, were above normal levels in 4 of 10 patients (Table 1), it is important to note that all patients included in this study had not yet developed liver cirrhosis. After flow cytometric sorting of γδ T cells, the highly diverse CDR3 regions of the TCR γ- and δ-chain were PCR amplified through gene-specific primers and subjected to NGS analysis (19). The workflow to analyze multiple samples from 14 healthy controls (HC) and 23 chronic HCV patients is depicted in Figure S1A in Supplementary Material. Flow cytometric analyses showed slightly decreased total γδ T cell frequencies, but a higher percentage of Vγ9^+^ T cells, in patients with higher ALT levels when compared with HC and chronic HCV patients having low ALT levels (Figures 1A,B). NGS of functional Vγ or Vδ chain usage of TCR repertoires (Figures 1C,D) further indicated almost no difference of V-chain distributions between HC and chronic HCV patients. Of note, box plots in Figure 1D suggest that at least some of the chronic HCV-infected patients had higher peripheral Vδ3^+^ frequencies and this was independent of ALT levels. To characterize γδ T cell repertoires in more detail, we analyzed the clonal distribution and diversity of TRG and TRD repertoires (Figures 1E,F). As observed in previous datasets of HC (19, 44, 45) and as illustrated by three HC of this study and six representative chronic HCV patients with different ALT levels, the 20 most expanded clones collectively made up between 25 and 60% of whole TRG or TRD repertoires (Figures 1E,F). Thus, γδ TCR repertoires of chronic HCV patients largely resembled HC and were highly diverse with few expanded clones, that are not necessarily Vγ9JP^+^ and Vδ2^+^ (Figures 1E,F). All datasets were summarized by depicting the median frequency of top 20 clones and median Shannon index, a parameter used to measure TCR repertoire diversity, and showed no significant differences between the given groups (Figures S2A,B in Supplementary Material). Together, these NGS results indicate that peripheral TRG and TRD repertoires of healthy persons and chronic HCV patients have a similar complexity and clonal composition.

**Table 1:** Baseline characteristics of healthy individuals and both cohorts of chronic HCV patients.

	Healthy	Chronic HCV (longitudinal samples)	Chronic HCV (one time-point)
*n* (m/f)	9 (4/5), 5 (3/2)	10 (5/5)	14 (8/6)
Age (years)	41 (26–51), 44 (21–66)	54 (47–60)	54 (25–79)
HCV RNA (IU/mL)		2,913,000 (140,000–6,700,000)	1,893,000 (76,000–6,300,000)
HCV genotype		1	1
ALT (U/L)		96.2 (51–289)	65.4 (22–138)
AST (U/L)		54.6 (24–108)	52.3 (24–108)
gGT (U/L)		55.9 (21–107)	108.6 (14–558)
Fibroscan (kPa)		7.4 (5.4–12.3)	7.9 (2.2–24.3)
Abs. lymphocyte count		2,150 (1,600–3,300)	2,121 (1,200–3,200)

*ALT, alanine aminotransferase; AST, aspartate aminotransferase; gGT, gamma-glutamyl transpeptidase; HCV, hepatitis C virus*.

### DAA Drugs Lead to Minor Changes on γδ T Cell Numbers

**Figure 2:**
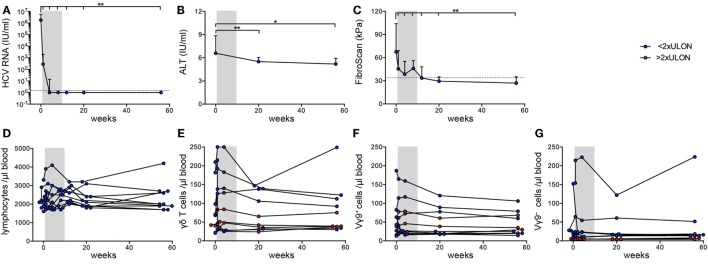
Clinical parameters and γδ T cell numbers in chronic hepatitis C virus (HCV) patients receiving DAA therapy. Graphs represent **(A)** HCV RNA levels in IU/mL and **(B)** alanine aminotransferase (ALT) levels in IU/mL, statistical analysis for both parameters with Wilcoxon test, as well as **(C)** fibroscan values in kPa, statistical analysis with paired *t*-test. **(A–C)** Median values with interquartile range from 10 chronic HCV patients before, during, and after DAA therapy are summarized. **(D)** Absolute lymphocyte counts were assessed for 10 chronic HCV patients at given time points. **(E–G)** The number of total **(E)**, Vγ9^+^ **(F)**, or Vγ9^−^ **(G)** γδ T cells per μL blood were determined from FACS data and lymphocyte counts shown for each of the 10 HCV patients individually. Gray shaded areas highlight the 8 weeks of DAA treatment. The number of total **(E)**, Vγ9^+^ **(F)**, or Vγ9^−^ **(G)** γδ T cells per μL blood were separated based on the ALT levels (red shows patients who have ALT levels >2 times ULON, blue indicates patients who have ALT levels <2 times ULON). Gray shaded areas highlight the 8-week DAA treatment.

Next, we investigated the effect of DAA-induced viral clearance on peripheral γδ T cell lymphocytes. Patients were treated for 8 weeks with a combination of sofosbuvir and ledipasvir. All patients achieved a sustained virological response (Figure S1A in Supplementary Material). Six of ten patients were virus-negative at therapy week 4 (w4); HCV RNA levels of the other four patients were already very low at this time point (<20 IU/mL) (Figure 2A). Some patients had mild liver fibrosis indicated by fibroscan values ranging from 5.4 to 12.3 kPa, which decreased significantly from therapy start (TS) to follow-up week 12 (fu12) (Table 1; Figure 2B). Some parameters for liver inflammation, such as ALT and AST levels, were slightly increased at TS (Table 1; Figure 2C), while bilirubin values were ranging in the normal level below 17 µmol/L (3.0–14.0 µmol/L) (Table 1). During DAA therapy, ALT levels decreased significantly within the first therapy week and reached normal levels at week 4 (Figure 2C). After initiation of DAA therapy, a slight increase of absolute lymphocyte numbers and γδ T cells/μL blood was observed (Figures 2D,E), which declined then until follow-up week 12 (Figure 2D). Furthermore, small changes in the number of Vγ9^+^ and Vγ9^−^ γδ T cells/μL blood were detected during the first therapy weeks while staying stable during the follow-up year (Figures 2F,G). Of note, patients with ALT values higher than twofold ULON had very low numbers of Vγ9^−^ γδ T cells/μL blood (Figure 2G), whereas the number of Vγ9^+^ γδ T cells/μL blood and their progression was similar to patients with ALT values lower than twofold ULON (Figure 2F). Altogether, patients improved with regard to liver inflammation and stiffness following successful eradication of HCV infection, while total numbers of Vγ9^+^ and Vγ9^−^ γδ T cells remained highly stable after viral clearance. This suggests that potential alterations in γδ T cell numbers and repertoire composition in response to HCV infection were sustained for the observation period of 48 weeks.

### TRD Repertoires of Vγ9^+^ and Vγ9^−^ γδ T Cells Are Distinct

**Figure 3:**
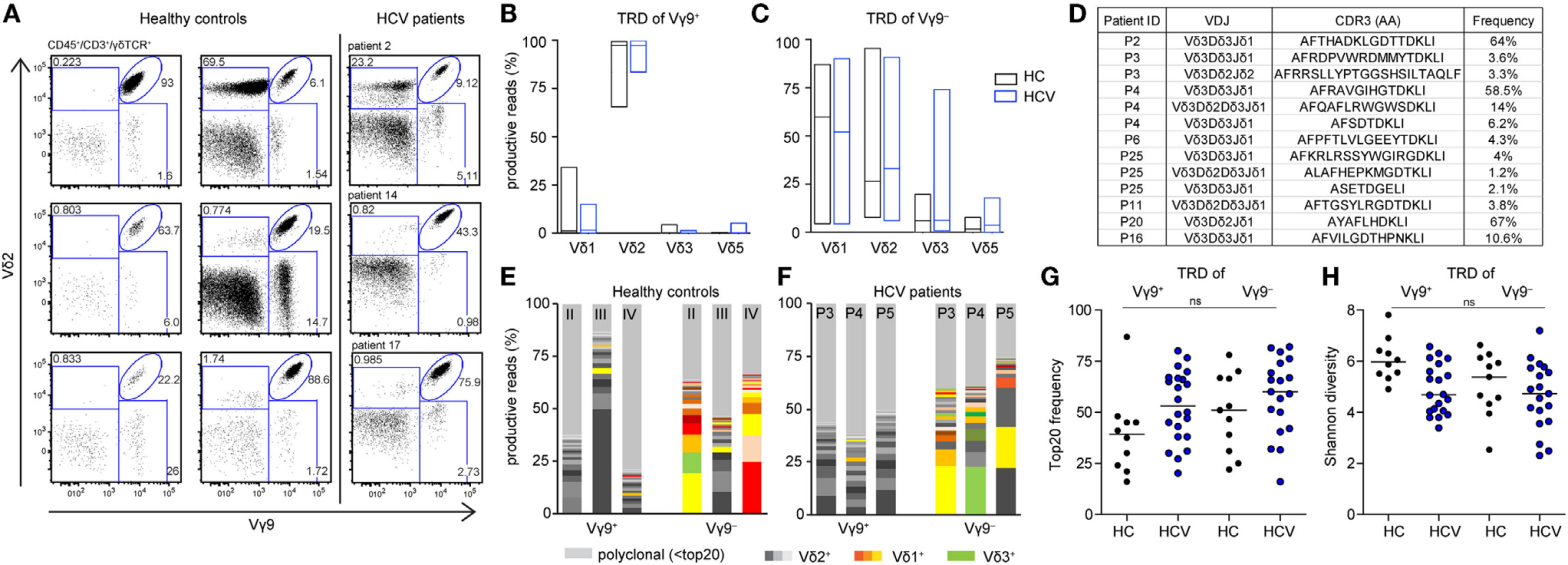
Differences between TRD repertoires of sorted Vγ9^+^ and Vγ9^−^ cells in healthy controls (HC) and chronic hepatitis C virus (HCV) patients. **(A)** Flow cytometric analysis of total γδ T cells for expression of Vγ9 and Vδ2 in six HC and three chronic HCV patients. **(B–H)** TRD next-generation sequencing analysis of sorted Vγ9^+^ and Vγ9^−^ γδ T cells from 11 HC and 23 HCV patients. **(B,C)** Fractions of V-delta chains within the sorted **(B)** Vγ9^+^ and **(C)** Vγ9^−^ populations of HC and HCV patients are represented as floating bars from minimum to maximum with the line representing the median. **(D)** Identified Vδ3^+^ sequences within sorted Vγ9^−^ cells of chronic HCV patients. **(E,F)** Top 20 expanded TRD clones of either Vγ9^+^ or Vγ9^−^ cells are plotted in stacked area graphs for **(E)** three HC or **(F)** three HCV patients. Vδ2^+^ are depicted in gray, Vδ1^+^ clones in orange, and Vδ3^+^ clones in green. Light gray represents non-top 20 TRD clones. Data sets were normalized to the percentage of productive reads per sample. **(G)** Dot plot displays the proportion of top 20 TRD clones within the Vγ9^+^ or Vγ9^−^ γδ T cell compartments for each analyzed sample. **(H)** TRD repertoire diversity of either Vγ9^+^ or Vγ9^−^ analyzed samples were determined by Shannon diversity indices. Samples were normalized to 10,000 productive reads. Median values are depicted as horizontal lines. Statistical analysis was performed using one-way ANOVA.

Next, we investigated Vγ9^+^ and Vγ9^−^ cells separately. Flow cytometric profiling of γδ T cells specified a fraction of Vγ9^−^Vδ2^+^ cells in the peripheral blood of adults and chronic HCV patients (Figure 3A), which is in line with the identification of expanded Vγ9^−^Vδ2^+^ clones in HC and transplant recipients (19). In adults, TRD repertoires are highly individual, while all TRG repertoires comprise public Vγ9JP rearrangements shared between all persons (19, 45). Here, we analyzed TRD repertoires of sorted Vγ9^+^ and Vγ9^−^ γδ T cells from 11 HC and 20 chronic HCV patients receiving DAA therapy (Table 1; Figure S1 in Supplementary Material). First, we studied the distribution of δ-chains within the Vγ9^+^ and Vγ9^−^ subsets in HC and chronic HCV patients. As depicted by boxplots (Figures 3B,C), Vγ9^+^ cells paired mainly with Vδ2^+^ sequences, while Vγ9^−^ cells paired with Vδ1, Vδ2, and Vδ3 sequences. In addition, the proportion of Vγ9^−^Vδ3^+^ cells was on average slightly increased in chronic HCV patients when compared with HC (Figures 1D and 3C) and could be associated with the identification of some expanded Vγ9^−^Vδ3^+^ clones (Figure 3D). Next, we analyzed the clonal distribution of the 20 most expanded TRD clones of Vγ9^+^ and Vγ9^−^ sorted cells from three representative HC (Figure 3E) and three chronic HCV patients (Figure 3F). Similar to total γδ T cell populations, TRD repertoires of Vγ9^+^ and Vγ9^−^ cell subsets were diverse and depicted an oligoclonal expansion of particular Vγ9^+^Vδ2^+^, Vγ9^−^Vδ2^−^, and Vγ9^−^Vδ2^+^ clones (Figures 3E,F). Comparison of the combined median frequency of the top 20 Vγ9^+^ and Vγ9^−^ TRD clones between HC and chronic HCV patients pointed to lower frequencies of top 20 clones in Vγ9^+^ cells of the HC group (Figure 3G). However, individual TRD repertoires were very diverse in the analyzed groups and thus differences in top 20 frequencies (Figure 3G) and TRD repertoire diversities as measured by Shannon indices (Figure 3H) were not statistically significant. Still, separate analyses of Vγ9^+^ and Vγ9^−^ sorted γδ T cells revealed that Vγ9^−^ TRD repertoires displayed a high Vδ-chain diversity and that expanded Vγ9^−^Vδ3^+^ clones existed in some chronic HCV patients, while the overall clonal composition of Vγ9^+^ and Vγ9^−^ TRD repertoires was comparable between healthy persons and chronic HCV patients. Further studies, preferably also including patients with more severe HCV disease, will be required to support or refute the hypothesis that Vγ9^−^Vδ3^+^ clones selectively expand in response to HCV infection.

### Stability of Vγ9^+^ and Vγ9^−^ TRD Repertoires During DAA Drug Therapy

**Figure 4:**
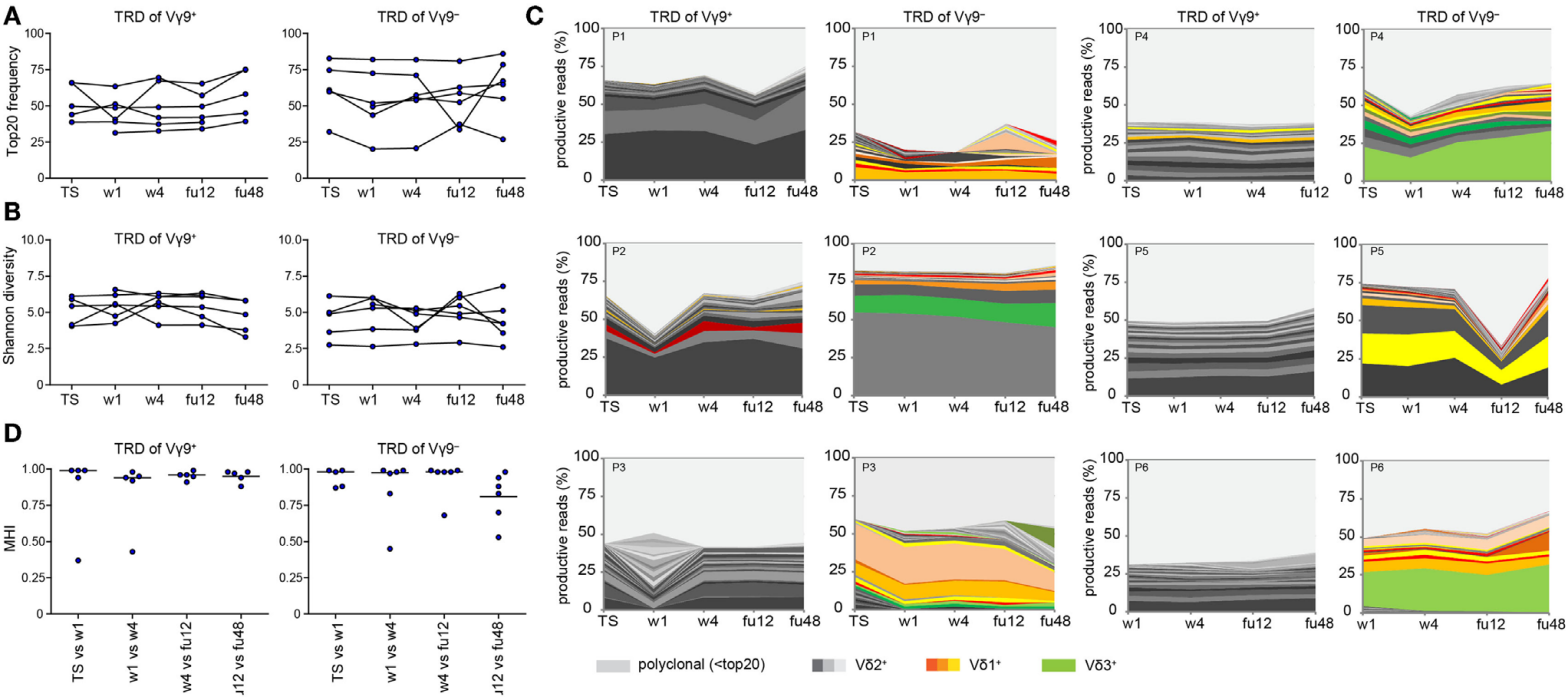
Monitoring of TRD repertoires of Vγ9^+^ and Vγ9^−^ γδ T cells in hepatitis C virus (HCV) patients over time. Next-generation sequencing results of TRD repertoires from sorted Vγ9^+^ or Vγ9^−^ cells of six chronic HCV patients receiving DAA therapy overtime (TS: therapy start; therapy week 1 and week 4: w1, w4; follow-up weeks: fu12, fu48). **(A)** TRD repertoire diversities were determined by Shannon values for Vγ9^+^ (left side) and Vγ9^−^ (right side) cells. Samples were normalized to 10,000 productive reads. **(B)** Frequency of top 20 TRD clones of sorted Vγ9^+^ (left side) and Vγ9^−^ (right side) cells. **(C)** Stacked area graphs illustrate the distribution of top 20 TRD clones within sorted Vγ9^+^ (left side) or Vγ9^−^ (right side) cells of chronic HCV patients from TS up to 1 year after DAA treatment (fu48). Vδ2^+^ clones are highlighted in gray, Vδ1^+^ clones in orange, and Vδ3^+^ clones in green. Light gray stands for non-top 20 TRD clones. Data sets were normalized to the percentage of productive reads. Horizontal lines represent median values. **(D)** To compare the TRD repertoire similarity between the given time points of either Vγ9^+^ (left side) or Vγ9^−^ (right side) cells, Morisita–Horn indices were calculated and visualized as dot plots. Zero means no overlap and one total overlap between all CDR3 sequences of two samples.

Finally, we asked whether chronic HCV infection and DAA-driven viral clearance would affect γδ T cell repertoires, which otherwise stay relatively stable over time (19, 44, 46). For this, we analyzed γδ TCR repertoires at TS, during DAA treatment and up to 1 year after therapy (illustrated in Figure S1A in Supplementary Material). We studied the TRD repertoires of sorted Vγ9^+^ or Vγ9^−^ cells as well as TRG and TRD repertoires of total γδ T cells. First, no significant changes in median frequencies of the top 20 clones and Shannon diversity indices of the analyzed cell subsets reflected that γδ TCR repertoires retained the overall complexity during the course of DAA drug therapy (Figures 4A,B; Figures S3A,B in Supplementary Material). Plotting the most expanded 20 TRD clones of either Vγ9^+^ or Vγ9^−^ sorted cells (Figure 4C) and the top 20 TRG and TRD clones of total γδ T cells (Figure S3C in Supplementary Material) over time, it turned out that only one patient (patient 3) showed notable changes in the distribution of expanded clones after TS. Importantly, this instability was caused by changes in Vδ2^+^ sequences of Vγ9^+^ sorted cells (Figure 4C). These might have been associated with increased γδ T cell counts starting from w1, but no other clinical parameters. Furthermore, γδTCR repertoire stability can be described in similarities between two given time points as calculated by Morisita–Horn indices. Notably, the Morisita–Horn index considers all clones of the given repertoire, while zero means no overlap and one represents complete overlap between all clones of given samples. Median values of calculated Morisita–Horn similarity indices revealed only minor changes of γδ T cell repertoires before, during, and after DAA drug therapy (Figure 4D; Figure S3D in Supplementary Material). In summary, γδ TCR repertoires of total, Vγ9^+^ or Vγ9^−^ γδ T cells retained their overall complexity during DAA therapy and were highly stable up to 1 year after viral clearance and normalization of liver enzymes.

## Materials and Methods

### Patient Characteristics

All 23 patients chronically infected with HCV as well as 9 HC were recruited at the Department of Gastroenterology, Hepatology and Endocrinology at Hannover Medical School, Germany. In addition, five HC were recruited at the Institute of Immunology/Department of Hematology, Hemostasis, Oncology and Stem Cell Transplantation at Hannover Medical School, Germany. The chronic HCV patients were analyzed over time before, during, and after novel DAA therapy for 8 weeks with a combination of sofosbuvir and ledipasvir. From 10 patients, peripheral blood mononuclear cells were collected at treatment start (TS), therapy week 1 (w1), therapy week 4 (w4), follow-up week 12 (fu12), and 1 year after treatment cessation (fu48), isolated and cryopreserved for deferred analysis. Further, 13 chronic HCV patients who were treated for 8 or 12 weeks with a combination of sofosbuvir and ledipasvir were included in the study with only one time-point during or after treatment to determine their TRD repertoires.

The ethics committee of Hannover Medical School approved this study (Study number: 2148-2014 and 2604-2014), and all patients provided written confirmed consent before enrollment. The clinical characteristics of the chronic HCV patients and the HC are summarized in Table 1.

### Flow Cytometric Analysis and Sorting

PBMCs were thawed, washed twice, and stained for 20 min at room temperature for flow cytometric analysis and cell sorting with the following antibodies: LIVE/DEAD Fixable Green Dead cell Stain Kit, Thermofisher or DAPI; CD14-FITC, clone M5E2, BD Biosciences; CD19-FITC, clone HIB19, BD Biosciences; γδ TCR-PE, clone 11F2, eBiosciences; Vγ9-PE-Cy5, clone IMMU 360, Beckman Coulter; Vδ2-APC, clone 123R4, Miltenyi; CD45-APC-Cy7, clone 5B1, Miltenyi; CD3-BV786 and CD3-PECy7, clone UCHT1, BD Biosciences. After staining, PBMCs were washed twice and stored on ice until acquisition and sorting on a FACSAria Fusion cell sorter (BD Biosciences). HC and patients recruited at the Department of Gastroenterology, Hepatology and Endocrinology were sorted for CD14^−^/CD19^−^ γδ T cells and HC recruited at the Institute for Immunology were sorted for CD45^+^/CD3^+^ γδ T cells. Flow cytometry data were analyzed using the Flow Jo software V.9.8 (Tree Star Inc., Ashland, OR, USA).

### TCR Amplicon Generation and NGS

For cDNA synthesis, extracted mRNA using the RNAeasy mini kit (Qiagen) of flow cytometric sorted Vγ9^+^ and Vγ9^−^ cells, 5 µL mRNA of both subsets was pooled equally for analysis of total γδ T cells. CDR3 TRG and TRD sequencing amplicons were generated as described previously (19), while using 25–30 PCR cycles. According to Illumina guidelines 96 samples were labeled with Nextera XT indices and subjected to Illumina MiSeq analysis using 500 cycle paired-end sequencing. 20% PhIX was added as an internal control and to increase library complexity. Illumina output fastq files were processed using ea-utils.

### TCR Repertoire Analysis

All sequencing files were annotated according to IMGT/High-Vquest. Only productive reads were taken into consideration for downstream processing of annotated sequences. Repertoire analyses were based on CDR3 amino acid sequences. Annotated V-chains were counted and CDR3 sequences were ranked according to their abundance to finally normalize the results to the percentage of productive reads within the given sample. All bioinformatics analysis was conducted using R (version 3.2.2) and bash shell commands. Shannon indices were calculated with the R library “vegan” and Morisita–Horn indices with VDJtools (47) and TcR (48). Analysis scripts are available upon request.

### Statistical Analysis

Data were analyzed using the GraphPad Prism version 6.0b and 4.0. To test for normal distribution of data, D’Agostino and Pearson omnibus normality test was applied. Normally distributed data of multiple or two groups was analyzed using the one-way ANOVA, paired *t*-test, or unpaired *t*-test depending on the data sets that were compared. Regarding non-normally distributed data, the Mann–Whitney test or Wilcoxon matched-pairs signed rank test was used.

### Data Availability

SRA files have been deposited at SRP128752.

## Discussion

In this study, we monitored peripheral γδ T cell repertoire dynamics in a homogenous cohort of chronic HCV patients before and during DAA therapy. All HCV patients had similar characteristics by being persistently infected with the HCV genotype 1, by no development of liver cirrhosis and by receiving a short 8 weeks therapeutic drug treatment. It was reasonable to expect an impact of chronic HCV infection and its clearance on γδ T cell dynamics, because γδ T cells play a role in the antiviral defense of CMV, HCV, and other viruses (22, 49, 50). During HCV infection, cytokine release (IFN-γ) by Vγ9JP^+^Vδ2^+^ and non-Vγ9JP^+^Vδ2^+^ may contribute to virus clearance, while HCV persistence is associated with low γδ T cell numbers and an impaired cytokine production (22). By contrast, chronic HCV patients with high liver inflammation rather have increased frequencies of cytotoxic γδ T cells when compared with healthy subjects or patients with no liver inflammation. In our study, all chronic HCV patients included had no or only mild liver fibrosis and mild liver inflammation, which might explain that they displayed no significantly, elevated numbers of γδ T cells. Importantly, eradication of the viral infection resulted in significant improvement of liver stiffness and inflammation as revealed by fibroscan values and ALT levels. Overall, our TCR analyses showed that uncomplicated chronic HCV infection and rapid viral clearance had only minor effects on the peripheral γδ T cell compartment, indicating that more drastic immunological events are required for perturbation of peripheral γδ T cell repertoires (19). However, we cannot exclude that TCR repertoires in the liver might be more affected by HCV clearance. Also, patients with liver cirrhosis may show more pronounced alterations of TCR repertoires during DAA therapy.

This study characterized, in addition to the analysis of total γδ T cell populations in peripheral blood, TRD repertoires of sorted Vγ9^+^ and Vγ9^−^ cells separately. As already demonstrated in preterm-infants and neonates, Vδ2 sequences pair mainly, but not exclusively with Vγ9JP^+^ TRG sequences (51). Flow cytometric results and TRD repertoire analysis of this study pointed out that Vγ9^−^Vδ2^+^ T cells are maintained until adulthood. While Vγ9^+^ cells displayed a more homogenous Vδ-chain usage (mainly Vδ2), TRD repertoires of Vγ9^−^ cells were more diverse and consisted of Vδ1^+^, Vδ2^+^, Vδ3^+^, but very little Vδ5^+^ sequences. Clonal distributions and the complete absence of any shared expanded TRD clones collectively illustrated that similar to all γδ T cells (19), Vγ9^+^ and Vγ9^−^ TRD repertoires are generally oligoclonal and individual. Although γδ TCR repertoires showed only minor alterations within patients with mild HCV infections, we identified several expanded Vγ9^−^Vδ3^+^ γδ T cell clones present in the peripheral blood of some chronic HCV patients. In general, Vδ3^+^ cells are highly enriched in the liver, but not in the blood, of healthy persons (23). Since in routine clinical practice liver biopsies are no longer performed in uncomplicated HCV infection and patient sampling starts only after diagnosis of persistent HCV infection, we can only speculate that these liver-specific Vδ3^+^ cells might undergo a HCV-induced clonal expansion to finally circulate in the peripheral blood of chronic HCV patients. Presence of NK cell markers and liver-homing markers (e.g., CD161) could strengthen the hypothesis Vδ3^+^ γδ T cell clones can be associated with liver specificity. In addition, it should be worthwhile to investigate a potential HCV-related antigen specificity of expanded Vγ9^−^Vδ3^+^ γδ T cell clones in future *in vitro* studies.

Another important finding of this study was the absence of significant and detectable effects of novel DAA therapy on γδ T cell frequencies and their TCR repertoires in peripheral blood. This is remarkable as the systemic inflammatory milieu shows profound changes already early during antiviral therapy—even though no complete restoration of various soluble inflammatory parameters occurs (40). The effect of spontaneous clearance of acute HCV infection and a longitudinal follow-up would be an appropriate control; however, those patients are rarely seen in the clinics. It is conceivable that γδ T cells might contribute to successful resolution of the disease. Nevertheless, the finding that peripheral γδ T cell compartments and their associated TCR repertoires were highly stable even 1 year after viral elimination is in line with previous observations for other cell types. This may suggest that distinct imprints on the immune system by long-lasting HCV infection can persist for years despite eradication of HCV, which may have clinical implications for some hepatic and extrahepatic disease manifestations. For instance, no changes in the short-term risk to develop hepatocellular carcinoma upon DAA treated were observed in the analyzed cohort of HCV patients (52). With regard to NK cells, it has been suggested that phenotypic and functional alterations during chronic HCV infections could be restored upon DAA therapy (53). NK cell phenotypes were altered upon IFN-free DAA treatment further resulting in modifications of the transcription factor profiles (54, 55). T cells have also been studied in HCV infection and during DAA-related viral eradication. The proliferative capacity of HCV-responsive CD8^+^ T cells could be restored in part (56) and a decrease in PD-1 expression on CD8^+^ T cells was observed upon successful DAA treatment (55). On the other hand, neither the frequency nor the phenotype of regulatory T cells was rescued upon viral clearance (57). Likewise, MAIT cells were reduced in frequency and their functions are affected by chronic HCV infection (58), and in particular peripheral MAIT cells could not be restored upon viral eradication (39, 40). All these studies were analyzing the phenotypic and functional changes of given immune cells by flow cytometry. Our data now contribute that the frequency of peripheral γδ T cell populations is neither affected by uncomplicated chronic HCV infection with no liver inflammation *per se* nor by rapid viral eradication upon DAA therapy. Likewise, conventional PEG-IFNα/Ribavirin therapy might not significantly change γδ T cell numbers; however, the presence of IFNα during this treatment regime may stimulate cytokine production by Vγ9^+^Vδ2^+^ (24, 34, 35). In this study, peripheral Vγ9^+^ and Vγ9^−^ cell TCR repertoires were largely undisturbed with regard to oligoclonality and TCR diversity by rapid viral clearance using IFN-free DAA therapies. During and after DAA treatment, peripheral γδ TCR repertoires displayed a high stability for up to 1 year, indicating that there is no dominant acute anti-HCV response of γδ T cells in patients with chronic HCV infection and also consistent with the assumption that chronic viral infection might leave a sustained footprint on the γδ T cell compartment in peripheral blood.

## Ethics Statement

The ethics committee of Hannover Medical School approved this study (Study number: 2148-2014 and 2604-2014), and all patients provided written confirmed consent before enrollment.

## Author Contributions

SR, JH, and VS conducted, analyzed, and interpreted experiments. CS-F recruited and organized healthy controls. SR and AD performed NGS. KD collected and organized HCV patient samples. CK, MC, HW, and IP discussed data and designed the study. SR, JH, HW, and IP wrote the manuscript.

## Conflict of Interest Statement

The authors declare that the research was conducted in the absence of commercial or financial relationships that could be construed as a potential conflict of interest.
