# Supplementary material for: Human γδ T Cell Receptor Repertoires in Peripheral Blood Remain Stable Despite Clearance of Persistent Hepatitis C Virus Infection by Direct-Acting Antiviral Drug Therapy
Source: Front Immunol. 2018 Mar 16;9:510. doi: 10.3389/fimmu.2018.00510 (PMC5864898; doi:10.3389/fimmu.2018.00510)
Supplement: Supplementary file 1 [file Image_1.PDF]

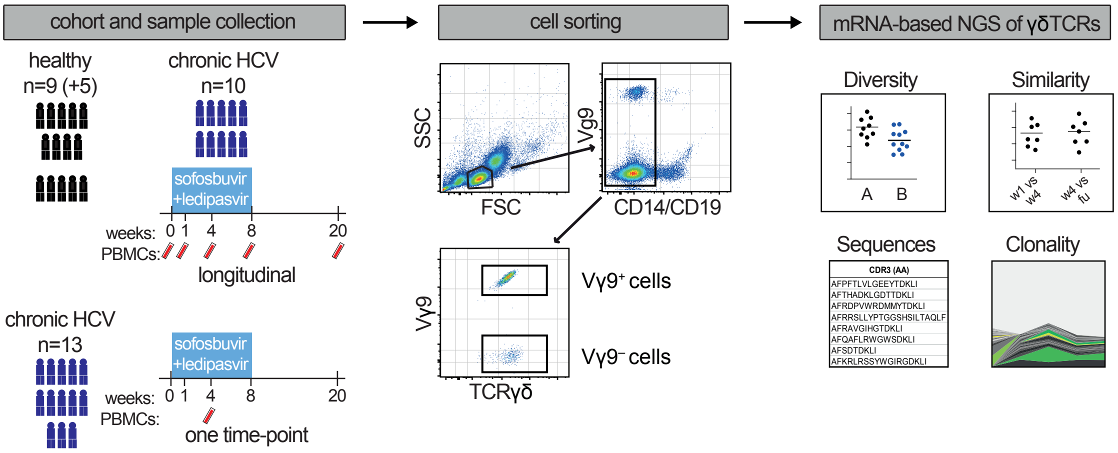

Supplementary Figure 1: Study design and TCR repertoire analysis of chronic HCV patients. (A) Overview of patient cohorts: healthy controls; 10 patients were monitored before, during and after DAA treatments; 13 patients were analyzed only at one time point during DAA treatment. After flow cytometric sorting (gating strategy) of Vγ9^{+} or Vγ9^{-} cells, samples were subjected to mRNA-based TRG and TRD repertoire analysis using NGS and bioinformatics.
