# Supplementary material for: Human γδ T Cell Receptor Repertoires in Peripheral Blood Remain Stable Despite Clearance of Persistent Hepatitis C Virus Infection by Direct-Acting Antiviral Drug Therapy
Source: Front Immunol. 2018 Mar 16;9:510. doi: 10.3389/fimmu.2018.00510 (PMC5864898; doi:10.3389/fimmu.2018.00510)
Supplement: Supplementary file 2 [file Image_2.PDF]

Supplementary figure 2

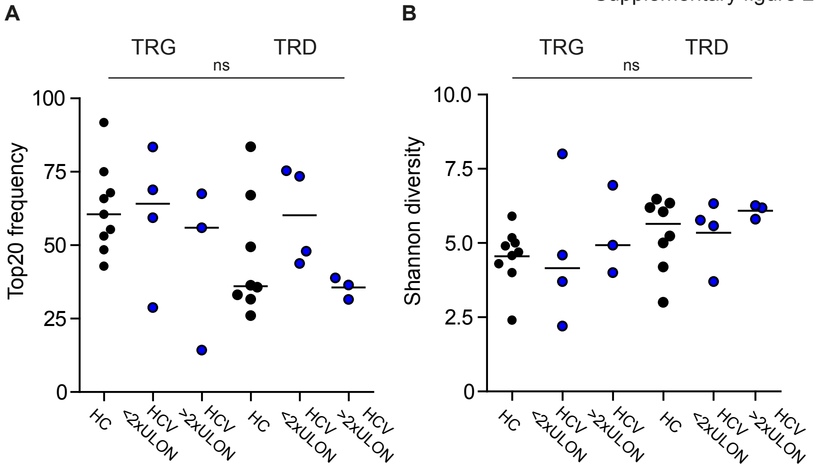

Supplementary Figure 2: Distribution of TRG and TRD repertoires in chronic HCV patients and healthy controls. (A) Dot plots present top20 clones frequencies or (**B**) Shannon diversity indices within TRG or TRD repertoires of eight healthy controls (HC, black dots) and seven chronic HCV patients (HCV, blue dots) grouped based on their ALT values. Four patients had ALT levels 2-times lower than upper limit of normal (<2xULON) and three patients had elevated ALT levels (>2xULON). For Shannon diversity analysis samples were normalized to 10000 productive reads. Horizontal lines indicate median values. One-way ANOVA was used for statistical testing.
