# Supplementary material for: Human γδ T Cell Receptor Repertoires in Peripheral Blood Remain Stable Despite Clearance of Persistent Hepatitis C Virus Infection by Direct-Acting Antiviral Drug Therapy
Source: Front Immunol. 2018 Mar 16;9:510. doi: 10.3389/fimmu.2018.00510 (PMC5864898; doi:10.3389/fimmu.2018.00510)
Supplement: Supplementary file 3 [file Image_3.PDF]

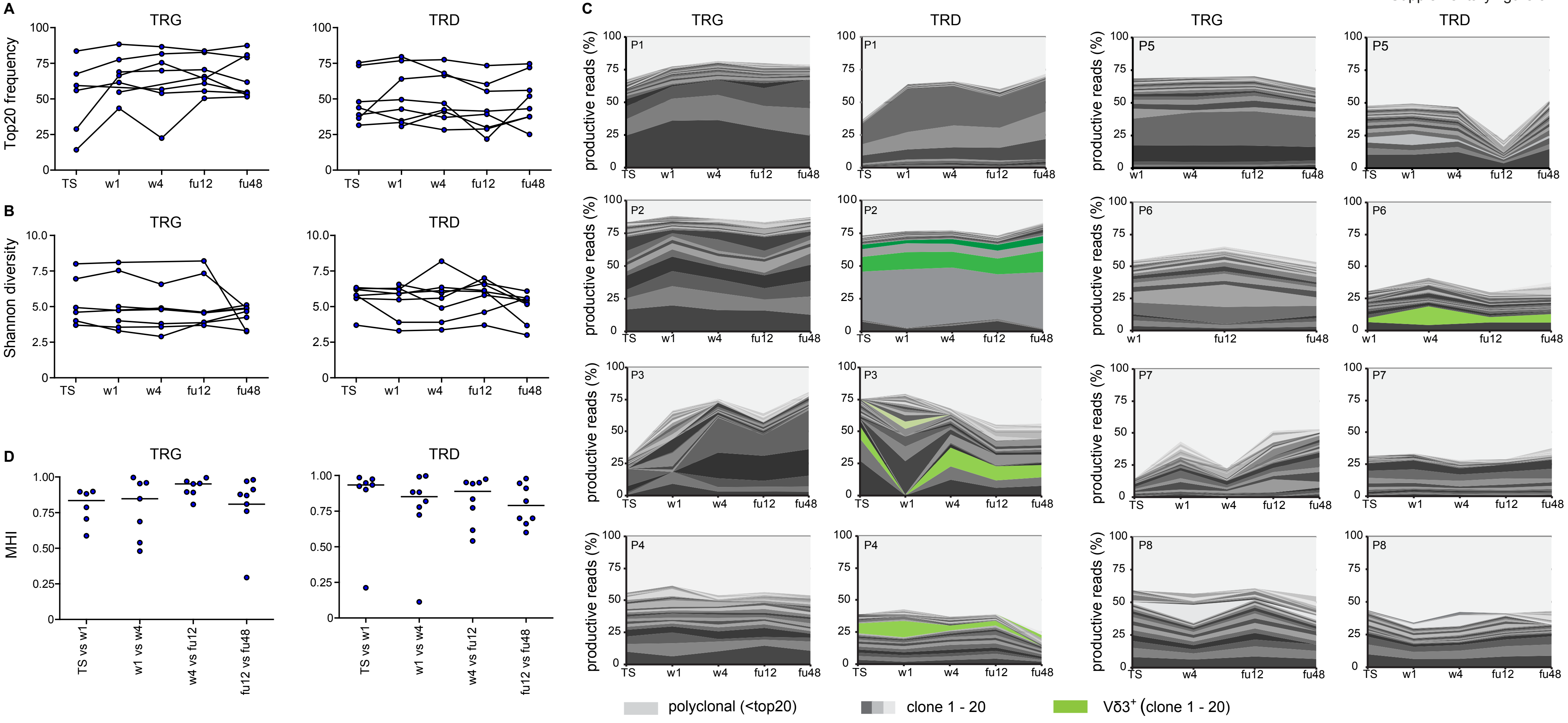

Supplementary Figure 3: Dynamics of TRG and TRD repertoires in HCV patients receiving anti-viral drug therapy. (A) Illustration of the frequencies of the 20 most expanded TRG (left side) or TRD (right side) in eight chronic HCV patients undergoing DAA therapy from therapy start (TS) until the follow-up (fu) week 48. **(B)** TRG and TRD repertoire diversities were addressed by Shannon values and monitored in chronic HCV patients before, during and after HCV clearance. Samples were normalized to 10000 productive sequence reads. **(C)** NGS of TRG and TRD analysis from total isolated γδ T cells of patients before, during and up to one year after DAA therapy. To visualize changes within either TRG or TRD repertoires from TS until the follow-up weeks, top20 clones are represented as stacked area graphs. All expanded top20 Vδ3^{+} clones are marked in green, all other top20 clones in grey colors and not expanded top20 clones in light grey. Data sets were normalized to the percentage of productive sequences. **(D)** Calculated Morisita-Horn similarity indices between the given time points are shown in dot plots. Horizontal lines depict median values, while zero indicates no overlap and one a total overlap of all clones.
